# Quantitative Magnetic Resonance Imaging of the Pancreas of Individuals With Diabetes

**DOI:** 10.3389/fendo.2020.592349

**Published:** 2020-12-04

**Authors:** John Virostko

**Affiliations:** ^1^Department of Diagnostic Medicine, University of Texas at Austin, Austin, TX, United States; ^2^Livestrong Cancer Institutes, University of Texas at Austin, Austin, TX, United States; ^3^Department of Oncology, University of Texas at Austin, Austin, TX, United States

**Keywords:** MRI, volume, relaxometry, perfusion, type 2 diabetes, type 1 diabetes, diffusion, radiomic

## Abstract

Magnetic resonance imaging (MRI) has the potential to improve our understanding of diabetes and improve both diagnosis and monitoring of the disease. Although the spatial resolution of MRI is insufficient to directly image the endocrine pancreas in people, the increasing awareness that the exocrine pancreas is also involved in diabetes pathogenesis has spurred new MRI applications. These techniques build upon studies of exocrine pancreatic diseases, for which MRI has already developed into a routine clinical tool for diagnosis and monitoring of pancreatic cancer and pancreatitis. By adjusting the imaging contrast and carefully controlling image acquisition and processing, MRI can quantify a variety of tissue pathologies. This review introduces a number of quantitative MRI techniques that have been applied to study the diabetic pancreas, summarizes progress in validating and standardizing each technique, and discusses the need for image analyses that account for spatial heterogeneity in the pancreas.

## Introduction

Recent advances in magnetic resonance imaging (MRI) technology have improved pancreas imaging, surmounting some of the difficulties inherent to the small, irregular shape of the pancreas, and its challenging location and susceptibility to motion. MRI of the pancreas has emerged into a valuable clinical tool for characterizing a number of pancreatic diseases. The staging and diagnosis of both acute (1) and chronic (2) pancreatitis often includes MRI. Furthermore, MRI can detect focal pancreatic lesions, and is employed clinically in both diagnosis of pancreatic cancer (3) and monitoring of therapeutic response (4). However, to date, MRI of the pancreas has had relatively limited impact on the study or management of diabetes. This likely stems in part from the established paradigm that diabetes effects only the endocrine pancreas, or islets, which are too small to be imaged by MRI. However, the involvement of exocrine pancreas in T1D and T2D is of renewed interest, as reviewed by Alexendra-Heymann et al. (5). As glucose, hormones, and other measures of glycemic control can be assessed using blood or urine tests, there may be an assumption that imaging is unnecessary. Indeed, two fields in which imaging has entered standard clinical practice, neurology and oncology, are both characterized by difficulty in directly assaying the tissue of interest and/or the absence of blood biomarkers. But while blood tests will likely always be the cornerstone of diabetes management, they are subject to important limitations. For instance, testing of A1C is hampered by standardization issues, poor sensitivity, and a lack of correlation with some pathophysiological hallmarks of diabetes (6). In type 1 diabetes (T1D), fasting hyperglycemia presents only after destruction of a majority of beta cells (7), suggesting that blood tests are a lagging indicator of beta cell loss due to a high insulin reserve. Circulating autoantibodies signify risk for T1D, but their presence can be transient and time to progression after autoantibody presentation is highly variable (8). These limitations and other aspects of diabetes care currently not well characterized by blood tests may be addressed in part by medical imaging.

This review focuses on quantitative MRI techniques and their application to study the pancreas in both type 1 and type 2 diabetes (T2D). These techniques are summarized in **Table 1**. Quantitative MRI refers to the objective measurement of parameters derived from digital images that characterize tissue attributes (9). This contrasts with the traditional qualitative assessment typically performed in standard radiology practice. Importantly, quantitative MRI techniques can map the entire pancreas, and thus identify regions of the pancreas with altered properties. This ability to interrogate spatial variation is significant, as histological studies have shown that the insult characteristic of both T1D (10) and T2D (11, 12) can differ across the pancreas. Furthermore, quantitative MRI parameters can be standardized across multiple sites for incorporation into clinical trials, and ultimately used to guide clinical practice. However, in order for quantitative MRI to be adopted to study the diabetic pancreas, the biological underpinnings of imaging results must be validated against gold standard measurements and techniques must be repeatable and reproducible across imaging centers. This review highlights a range of quantitative MRI parameters that may improve our understanding of the diabetic pancreas, benchmarks each technique’s progress in validation and standardization, and concludes with a discussion of image analyses that can accurately characterize pancreas heterogeneity.

**Table 1:** Quantitative magnetic resonance imaging (MRI) techniques.

MRI Technique	Pancreas Pathology Interrogated	Change in T1D	Change in T2D	Biological Confounders	Technical Confounders
**Anatomical MRI**	Pancreas Volume	↓↓	↓	Age; weight	
**Fat Fraction Mapping**	Fat infiltration	↔	↑	Age, sex, visceral adiposity	MRI acquisition; pancreas heterogeneity
**Longitudinal Relaxation (T1)**	Fibrosis	?	?	Age	MRI acquisition; magnetic field strength; pancreas heterogeneity
**Extracellular Volume Fraction (ECV)**	Fibrosis	?	↑		MRI acquisition; contrast agent administration; magnetic field strength; pancreas heterogeneity
**Diffusion-Weighted Imaging (DWI)**	Cell density; membrane integrity; fibrosis	↑	?	Age, sex	MRI acquisition; magnetic field strength; pancreas heterogeneity
**Dynamic Contrast-Enhanced (DCE)**	Perfusion; vascular permeability	?	↑		MRI acquisition; contrast agent administration; processing technique; pancreas heterogeneity
**Arterial Spin Labeling (ASL)**	Perfusion	?	?		MRI acquisition; pancreas heterogeneity
**Incoherent Intravoxel Motion (IVIM)**	Microvascular perfusion	?	?		MRI acquisition; processing technique
**Blood Oxygen Level Dependent (BOLD)**	Perfusion; oxygen consumption	?	?		
**Magnetic Resonance Elastography (MRE)**	Stiffness; fibrosis	?	↑	Age	MRI acquisition; processing technique

↑ = increase; ↓ == decrease; ↔ = no change; ? = unknown.

## Pancreas Volume

**Figure 1:**
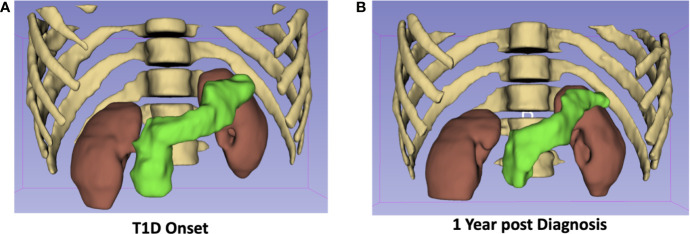
The pancreas of a 19-year-old male at diagnosis with T1D **(A)** is 25% smaller one-year post diagnosis **(B)**. The pancreas is displayed in green with the kidneys, ribs, and spine shown for anatomical context.

Owing to its exquisite soft tissue contrast, MRI can delineate pancreas borders from neighboring organs, which can in turn be used to quantify pancreas size. The pancreas has long been known to be smaller in type 1 diabetes (13) and more recently in type 2 diabetes as well (14). A meta-analysis validated these findings across multiple studies and found smaller pancreas size in T1D than T2D (15). Of note, the degree of pancreas reduction in both type T1D and T2D far exceeds the volume of the endocrine pancreas, suggesting exocrine involvement in diabetes pathogenesis. These imaging findings are supported by histological studies of the pancreas from T1D donors which found reduced numbers of acinar cells (16) and altered exocrine cell expression (17) versus controls. Investigations into the temporal dynamics of pancreas size in T1D have found reduced pancreas size at diagnosis (18, 19), in individuals at risk for disease (20, 21), and longitudinal declines over the course of disease (20). Example images demonstrating the longitudinal decline seen in pancreas size in one individual with T1D is shown in **Figure 1**. Taken together, these studies suggest that alterations in pancreas size may be an early hallmark of T1D risk and may correlate with disease progression.

The relationship between pancreas volume and beta cell mass or function remains unclear. An islet transplantation study found a direct correlation between pancreas size and islet yield, and that this relationship was dependent on donor HbA1c (22). This suggests that pancreas size may reflect a combination of islet mass and function. Pancreas volume measurements performed using MRI display high accuracy, repeatability, and reproducibility. Porcine pancreas volume calculated from MRI demonstrated excellent correlation with gold standard water displacement measurements (23), indicating the accuracy of the technique. Furthermore, in human subjects who received MRI scans in quick succession, measurements of pancreas volume were highly repeatable (24, 25). Pancreas borders can be difficult to delineate, which may lead to reader subjectivity in pancreas volume measurements. However, reproducibility across multiple readers reveals good agreement between two readers outlining the same image (24, 25). One caveat is that pancreas volume changes over the course of the human lifespan in the absence of disease, with rapid increases in size over childhood and pancreas atrophy in later life (14). Current quantification of pancreas size typically normalizes to body weight or surface area to account for these dynamics in pancreas size over the lifespan. Other factors that account for the large variations in pancreas volume seen between individuals are currently not known, but warrant further investigation.

## Fat Fraction Mapping

**Figure 2:**
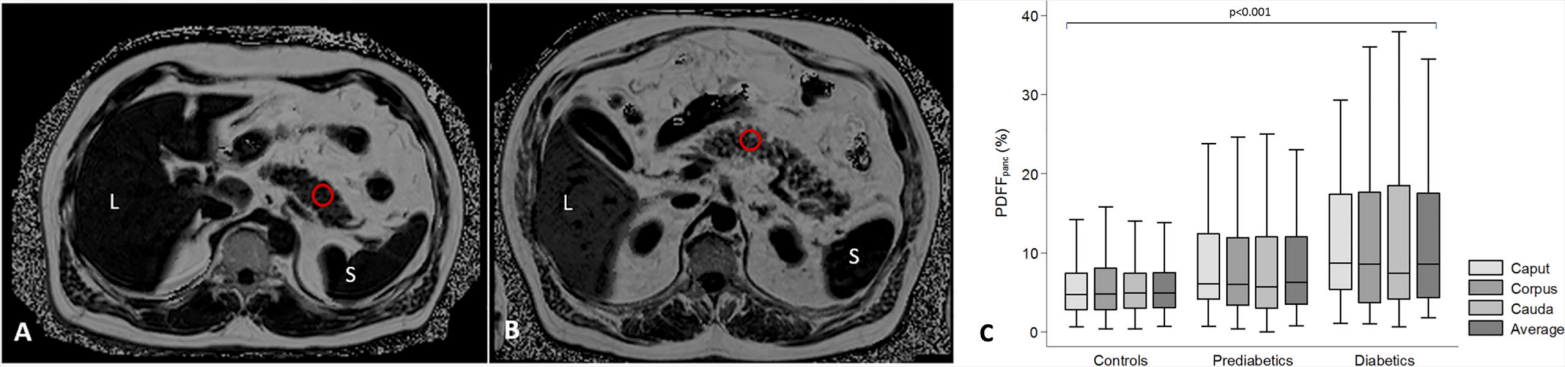
Assessment of pancreatic fat content in subjects with lower **(A)** and higher **(B)** pancreatic fat content. Pancreatic fat content was measured as proton-density fat fraction (PDFF_panc_) in a region of interest (red circle). L = liver; S = spleen. **(C)** Differences of pancreatic fat content between controls, subjects with prediabetes and diabetes displayed by box-and-whisker. Figure adapted from Heber et al. (32) under a Creative Commons License.

MRI can measure the fat composition of tissue, as protons associated with fat and water spin with slightly different frequencies. Thus, their relative concentrations in a voxel (a portmanteau of “volume element”, i.e., a three-dimensional pixel) of interest are relatively easy to separate. The relationship between pancreatic fat deposition and diabetes has been demonstrated in rodent models, in which ectopic fat accumulation leads to beta cell dysfunction (26). Similarly, MRI studies of people with T2D have demonstrated higher pancreatic fat associated with reduced beta cell function (27, 28). However, a number of other studies have failed to find a correlation between T2D and pancreatic fat content (29, 30). MRI may be sensitive to declines in pancreatic fat content. A study of diet-induced reversal of T2D found that diabetes reversal was accompanied by a decline in pancreatic fat fraction (31). Furthermore, pancreatic fat content may be increased in individuals at risk for developing T2D (32, 33) (**Figure 2**). In contrast with studies of T2D, MRI measures of pancreatic fat content are not altered in T1D (34), although some autopsy studies have found fatty infiltrate in the pancreas of T1D donors.

In terms of standardization and validation, fat fraction measurements are relatively advanced, owing in part to the simplicity of the technique and the availability of standard processing tools from MRI vendors. Histological measures of fat content from resected pancreata and MRI quantification of fat fraction demonstrate excellent agreement (35). Furthermore, test-retest measurements of pancreatic fat content calculated using MR spectroscopy were found to be repeatable (36). However, a meta-analysis of five studies of pancreatic fat content in T2D demonstrated high heterogeneity in fat measurements between studies (15). As is typical of MRI techniques, the acquisition and processing used to calculate the fat fraction may impact the measurement (37), and may account for some of the differences seen between studies. Additionally, there exist regional variations in fat fraction throughout the pancreas, with different values in the head, body, and tail (38) (**Figure 2**). Finally, pancreatic fat content can be influenced by age, sex, and visceral adipose tissue (32). These demographic factors must be carefully accounted for when examining pancreatic fat content in individuals with diabetes. Unfortunately, reference standards for pancreatic fat content and its relationship with demographic factors are not well established at present.

## Relaxometry

### Longitudinal Relaxation (T1)

**Figure 3:**
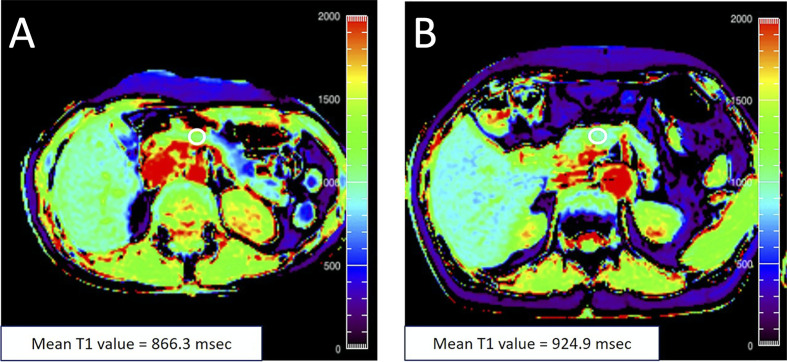
**(A)** A 48‐year‐old female with an HbA1c value of 5.5% with T1 map displayed in rainbow color shows the mean pancreatic T1 value of 866.3 msec. **(B)** A 76‐year‐old female with an HbA1c value of 6.0% with T1 map displayed in rainbow color shows the mean pancreatic T1 value of 924.9 msec. The white circle in each panel indicates a regions-of-interest (ROI) manually placed in the pancreas. Figure adapted from reference (44) with permission, © 2020 John Wiley & Sons, Inc.

Generation of an MR image relies on perturbation (or tipping) of protons from the main magnetic field by a radiofrequency pulse. Longitudinal relaxation refers to the return of these perturbed protons to their equilibrium state. The relaxation rate for each voxel within an image is a function of the intrinsic tissue parameters and is quantified by the time constant T1 (not to be confused with T1D). This is the basis for so called T1-weighted images, in which fat, injected contrast agents, and tissues with high protein content appear bright. Of note, the pancreas appears bright on a T1-weighted image, presumably due to the presence of high levels of aqueous protein in the acinar cells (39). In addition to qualitative T1-weighted images, one can employ a variety of techniques to quantify the T1 value characteristic of each voxel (40). Quantitative T1 mapping of the pancreas has demonstrated increased T1 in chronic pancreatitis (41), suggested that T1 may be sensitive to pancreatic inflammation. In studies of individuals with T2D, the T1 value was found to be higher than controls and correlated with HbA1c in one study (42). In contrast, another study found lower T1 values in T2D, although these values were also significantly different than controls (43). The T1 of the pancreas in individuals with prediabetes is also increased (44) (**Figure 3**), suggesting that the alterations responsible for prolonged longitudinal relaxation may occur early in the development of T2D.

Validation and standardization of T1 measurements of the pancreas are underway. The foremost hurdle is determining the specific pancreas pathology reflected by T1 measurements. In the heart, histological measurements of fibrosis have correlated with T1 (45). Similar studies of resected pancreas are limited but have shown correlation of T1 with grade of pancreatic fibrosis (46). Standardization of T1 measurements must take into account known correlations between pancreas T1 values and age (47), although these associations have not been seen in all studies (48). Additionally, there is a known dependence of T1 on magnetic field strength of the MRI scanner (47). Finally, there are a variety of different techniques used to generate T1 maps. These methods display some discrepancies when applied to image the pancreas of the same individual (49). Further work is needed to standardize T1 mapping techniques for application to the pancreas.

### Extracellular Volume Fraction (ECV)

Contrast agents are often used to alter tissue enhancement on MR images. These contrast agents are typically paramagnetic agents which shorten T1, thus leading to enhancement of tissues with high contrast agent perfusion. This technique is commonly used in oncological imaging, where well vascularized tumors “light up” after contrast agent administration. By examining changes in T1 maps in both the tissue of interest and blood and coupling it with the hematocrit, the extracellular volume fraction (ECV) can be calculated. ECV has been developed primarily for cardiac MRI applications, and has been found to be sensitive to a number of cardiomyopathies (50). In the pancreas, ECV has been shown to increase with increasing grade of chronic pancreatitis (51). ECV of the pancreas is also higher in individuals with T2D and, similar to native T1 maps, correlates with HbA1c (42).

The biological basis for increased ECV in the pancreas in diabetes is assumed to be due fibrosis. However, while MRI has demonstrated correlation between fibrosis and ECV in the heart (52), similar correlation of imaging and pathology are lacking in the pancreas. ECV values in the pancreas do not appear to be influenced by age or sex (47). The repeatability and reproducibility of ECV measurements have been established in the myocardium. These studies have demonstrated general agreement, although as with non-contrast enhanced T1 measurements, the technique used for quantification can impact the result (53). Of note, ECV requires administration of a contrast agent containing gadolinium, which has been the subject of recent concerns over long term brain retention.

## Diffusion-Weighted Imaging (DWI)

**Figure 4:**
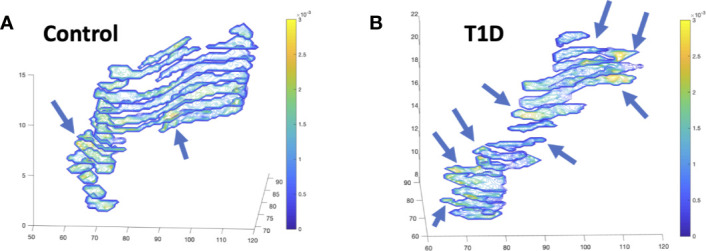
Diffusion-weighted imaging (DWI) of the pancreas of a control **(A)** and individual with recent-onset T1D **(B)** displays focal alterations in the apparent diffusion coefficient (ADC), designated with arrows, with more focal areas of increased diffusion in T1D. Images display contiguous slices spanning the pancreas pseudo colored with contour plots according to the ADC value according to the ADC value in units of mm^2^/s.

Diffusion-weighted imaging (DWI) measures the random Brownian motion of water molecules within a voxel. In biological tissue, DWI reflects an amalgamation of cell density, cell membrane integrity, and viscosity. DWI can be quantified by adjusting the diffusion weighting (commonly referred to as the b-value) of two images and comparing their intensities to yield the apparent diffusion coefficient (ADC). While early applications of DWI were primarily limited to the brain due to long imaging times, advances in MRI hardware and processing have reduced acquisition times for abdominal imaging. In the pancreas, MRI has proven useful for detecting and characterizing malignant pancreatic masses (54), similar to the success of DWI in other oncological applications. Furthermore, in chronic pancreatitis the ADC value has been found to be reduced compared with controls (55). Applications of DWI to study the diabetic pancreas are limited. One study found reduced ADC in individuals with fulminant T1D (55). Our study of individuals with recent onset T1D did not find differences in ADC versus controls when measurements were averaged throughout the pancreas (20). However, we did find altered distributions of ADC in the pancreas in T1D, with an increased number of voxels with high ADC values in T1D. These areas of high ADC (corresponding to areas of increased water diffusion, and presumably inflamed tissue) were found in focal areas in the pancreas in T1D at a greater rate than control pancreas (**Figure 4**). In individuals with suspected pancreatic disease, a negative correlation was found between pancreatic ADC and HbA1c (56). In addition to ADC, DWI can be quantified using other metrics. For instance, diffusion kurtosis can be calculated by assuming non-Gaussian Brownian motion and acquiring DWI with strongly diffusion-weighted images (higher ‘b-values’). This kurtosis is thought to reflect the restriction of water diffusion by cell membranes and other tissue microstructure. Similar to ADC, diffusion kurtosis was found to correlate with HbA1c, with higher kurtosis in the group with highest HbA1c (56).

As DWI is a mixed measure that reflects a number of tissue parameters influencing water diffusion, the biological basis in the pancreas is still under investigation. One study of resected pancreas found reduced ADC in more fibrotic tissue (46), presumably reflecting increased cellular density in fibrous tissue. The ADC in healthy pancreas appears to display regional variation over the pancreas, with highest values in the pancreas head and lowest values in the body (57). ADC values are also influenced by age and sex (58). A study across multiple MRI scanners found that ADC measures of the pancreas are generally reproducible, although, similar to T1 measurements, ADC is influenced by magnetic field strength (59). Measurement of ADC in the pancreas is likewise influenced by the acquisition scheme. DWI acquired with higher diffusion weighting (higher b-values) results in a higher calculated ADC (60). Thus, accurately quantifying DWI of the diabetic pancreas requires standardization of image acquisition and normalization for pancreas anatomy and patient demographics.

## Perfusion

The endocrine pancreas is characterized by a dense network of capillaries, which receive a significantly higher rate of blood flow than exocrine tissue (61). Alterations in islet vasculature have been found in the pancreas of organ donors with both T1D (62) and T2D (63). Thus, there is intense interest in studying islet blood flow and measuring it non-invasively in humans. MRI is widely used clinically to measure cerebral and myocardial perfusion. Four different MRI techniques for measuring perfusion are introduced below along with implications for pancreas imaging.

### Dynamic Contrast-Enhanced MRI (DCE-MRI)

**Figure 5:**
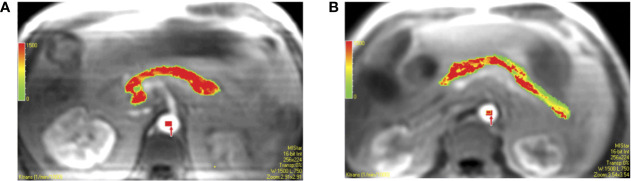
Pixel-by-pixel color maps for transfer constant obtained with region of interest analysis. Calculated values of each pixel in region of interest can be seen in colors (red, yellow, and green are high, middle, and low values, respectively). **(A)** Color map in 61-year-old male coronary artery disease patient with type 2 diabetes shows a mean transfer constant = 1.291 min^−1^. **(B)** Color map in 54-year-old male coronary artery disease patient without type 2 diabetes shows a mean transfer constant = 0.787 min^−1^. Figure reproduced from reference (67) with permission, © 2009 RSNA.

Dynamic contrast-enhanced MRI (DCE-MRI) relies upon acquisition of T1-weighted MR images after injection of a paramagnetic contrast agent. For further information on T1-weighting and MR contrast agents, please refer back to *Longitudinal Relaxation (T1)* and *Extracellular Volume Fraction (ECV)*, respectively. These contrast agents traverse through the vascular network and extravasate out of permeable vessels. The “dynamic” nature of this technique is derived from the acquisition of serial images over the time course of contrast agent distribution. The resultant time activity curves for each voxel in the image can be analyzed using pharmacokinetic models to yield estimates of perfusion parameters (64). DCE-MRI has shown promise for imaging pancreatitis (65) and tracking response of pancreatic cancer to therapy (66). A study of DCE-MRI in individuals with T2D found increased vascular permeability but lower plasma volume in the pancreas (**Figure 5**), and that the magnitude of these perfusion changes increased with longer disease duration (67). A later study was unable to replicate these findings, and also did not determine an effect of glucose bolus on DCE-MRI parameters (43).

Correlation of DCE-MRI parameters and pathology has been performed in pancreas sections resected from individuals with pancreatic cancer. This analysis found correlation between DCE parameters and both fibrosis and microvascular density (68). Repeatability and reproducibility of DCE-MRI has been notoriously difficult to both measure and improve, as measurements can be influenced by acquisition parameters, choice of pharmacokinetic model, and contrast agent administration protocol. One study of repeatability in the pancreas found variability of 21% and also measured regional variations in the pancreas head, body, and tail (69). As with ECV measurements, contrast agent safety can be a concern when imaging in young individuals or performing repeat scans.

### Arterial Spin Labeling (ASL)

Concerns over contrast agent injection have spurred the development of MRI techniques sensitive to perfusion that do not require exogenous sources of contrast. One such technique is known as arterial spin labeling (ASL) which magnetically labels blood flowing through a plane containing feeding blood vessels and images the resultant distribution of this tagged blood volume. ASL has demonstrated the ability to image increases in pancreas perfusion due to secretin (70), a hormone known to induce pancreatic fluid and bicarbonate secretion. Similarly, ASL of the pancreas has measured increased pancreas perfusion in response to glucose bolus (71). A study performing ASL during hyperglycemic clamp did not detect a difference between individuals with T1D and controls (72). However, ASL has been subject to a number of recent technological advances and continues to rapidly improve.

There have not been studies comparing ASL measurements in the pancreas to pathology. However, comparisons between DCE-MRI derived perfusion with those from ASL display strong agreement (73). Thus, ASL may also reflect microvascular density throughout the pancreas. Of note, this parameter may vary across the pancreas, as ASL measurements found differences in the pancreas heady, body, and tail (74). The repeatability of ASL in the pancreas is moderate (70). Further work is needed to define the reproducibility of ASL across different MRI scanners and establish standardized acquisition and processing schemes.

### Intravoxel Incoherent Motion (IVIM)

In previous discussion of diffusion-weighted MRI (Section 5), we noted that Brownian motion can be analyzed as having non-Gaussian distribution. While analyzing high b-value data yields diffusion kurtosis measurements, fitting non-Gaussian diffusion using low b-value data yields intravoxel incoherent motion (IVIM). IVIM is thought to be sensitive to the microscopic perfusion of capillaries (75). In pancreas imaging, IVIM may help characterize benign versus malignant pancreas lesions (76). While we are not aware of studies of IVIM in individuals with diabetes, IVIM has demonstrated correlation with glucose stimulated perfusion increases in porcine models (77). Future studies examining response to glucose in diabetes using IVIM are warranted.

The biological basis of IVIM in the pancreas has been demonstrated to derive from the blood component using blood suppression techniques (78). Furthermore, IVIM parameters agree with PET and microsphere measurements of pancreas perfusion (77). Repeatability and reproducibility of pancreas IVIM is influenced by both image acquisition and processing techniques (79). Currently, acquisition and processing of IVIM are complex techniques which are not well standardized across studies.

### Blood Oxygen Level Dependent (BOLD)

BOLD imaging is widely used in neuroscience to image brain activity. BOLD reflects the accumulation of deoxyhemoglobin in response to oxygen consumption (which in the brain is posited to be a surrogate for neural activation). Fewer BOLD applications have been performed outside the central nervous system, although there are studies exploring renal oxygenation using BOLD (80). Similarly to IVIM, studies in individuals with diabetes are lacking, but there is a report showing alterations in BOLD MRI in the pancreas after glucose ingestion (81).

Decades after its first use, the biological basis of BOLD MRI is still under investigation. The biological correlate of BOLD signal in the pancreas, as well as its repeatability and reproducibility are not well characterized.

## Magnetic Resonance Elastography (MRE)

**Figure 6:**
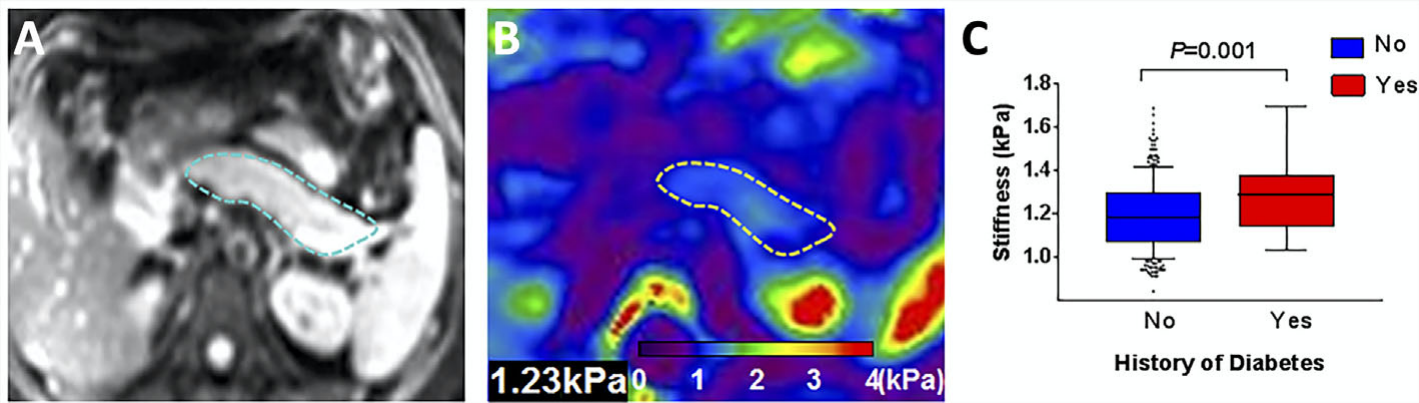
Representative pancreatic axial magnitude images (**A**, pancreas outlined in blue) and elastograms (**B**, pancreas outlined in yellow). Box plot in **(C)** compares pancreas stiffness in individuals with a history of diabetes versus controls. Figure adapted from reference (84) with permission, © 2020 John Wiley & Sons, Inc.

Magnetic resonance elastography (MRE) consists of imaging performed while a tissue is subjected to high frequency vibrations. MRE interrogates the movement of vibration-induced shear waves through the body; these waves move slower in stiffer tissues. The primary clinical application of MRE currently is detecting fibrosis and cirrhosis in chronic liver disease. Applications to the pancreas are limited by the small size of the pancreas, but have found increased stiffness in chronic pancreatitis (82). Similar to perfusion measurements, MRE has been performed after glucose bolus which led to pancreas stiffening (83). Additionally, individuals with diabetes have been found to have higher pancreas stiffness than controls (84), as shown in **Figure 6**.

Histological studies of resected pancreata found a correlation between MRE-derived stiffness and both fibrosis and acinar atrophy (85). MRE measurements of pancreas stiffness are repeatable in test-retest studies, but are dependent on the vibration frequency used (83) and the age of the individual (86).

## Radiomics: Accounting for Heterogeneity Throughout the Pancreas

MRI of the pancreas interrogates the complex structure of the pancreas encompassing islets, acinar cells, and the ductal network. There are known differences across the pancreas in the relative concentration of these components, with higher relative numbers of islet in the pancreas tail (87). A common theme throughout the MRI techniques introduced in this review is that they were frequently different in the pancreas head, body, and tail of the same individual. This was seen for measurements of fat fraction (38), diffusion (57), and perfusion measured by DCE (69) or ASL (74). Furthermore, spatial heterogeneity of the pancreas is further altered in diabetes, as has long been appreciated in autopsy studies of the pancreas from donors with diabetes (88). In T1D, there is marked spatial heterogeneity in the presence of the immune infiltrate characteristic of T1D (10) as well as individuals at risk for disease (89). Similarly, the islet amyloids and corresponding exocrine fibrosis found in T2D show lobular distribution throughout the pancreas (11, 12). The heterogeneity of quantitative MRI parameters throughout the pancreas is evident in **Figures 2**–**6**.

The spatial heterogeneity in MRI measures of the pancreas in general, and in the diabetic pancreas specifically, has important implications when quantifying MRI parameters. MRI analysis is commonly performed using a regions-of-interest (ROI) placed over a section of the pancreas, as demonstrated in **Figures 2** and **3**. However, in a mosaic tissue such as the pancreas the choice of ROI placement can influence the parameter of interest. An ROI placed in the same individual’s pancreas may have significantly different values if it is placed in the pancreas head versus tail. Even within the same region of the pancreas, ROI-based calculations may be dependent on the proportion of endocrine, ductal, or fatty infiltrate encompassed in the ROI. A helpful analogy can be found in histology, where analyzing only a single section of a slide induces sampling bias. Averaging measurements over multiple sections and slides gives more robust analysis of histological samples. Similarly, one can average MRI parameters over the entire pancreas, although this technique may be insensitive to sparse alterations, such as those found using DWI in T1D (20). For instance, if only a small portion of the pancreas is affected but the entire pancreas is averaged together, then the preponderance of similar intensity voxels can average out sparse voxels with significantly higher or lower intensities.

The insufficiency of ROI or whole organ-based analyses coupled with advances in high performance computing has led to so-called “radiomic” analysis of medical image data. The field of radiomics, excellently reviewed by Gillies et al. (90), couples high throughput feature extraction from quantitative imaging data with multi-dimensional texture, histogram, shape, and wavelet analysis. These radiomic features are uniquely suited to quantify spatially variant organs, such as the different habitats present in tumors (91). Likewise, radiomic analysis show great promise for characterizing and quantifying the spatial heterogeneity of the diabetic pancreas. For example, we have demonstrated that histogram analysis of the pancreas of individuals with T1D identifies differences in DWI that is not evident when averaging the whole pancreas (20). The relative advantages and disadvantages of image analysis techniques for pancreas MRI are summarized in **Table 2**. Radiomic analysis of the diabetic pancreas has not been well characterized and represents a promising new direction in the field.

**Table 2:** Pancreas magnetic resonance imaging (MRI) analysis techniques.

Technique	Advantages	Disadvantages
**Region-of-interest**	Easy to implement; fast; can avoid ductal structures and organ borders	Placement affects quantification; does not capture anatomical heterogeneity
**Whole pancreas averaging**	Not subject to reader placement; captures all regions of the pancreas	Averages ductal structures and organ borders; may average out sparse alterations; requires capturing entire pancreas in field of view
**Radiomics**	Quantifies anatomical heterogeneity; identifies anatomical regions with different properties (“habitats”)	Difficult to implement; High dimensionality of data can result in overfitting

## Conclusion

Quantitative MRI techniques have been rapidly integrated into clinical practice across a number of medical specialties. Their contribution to endocrinology and the study of the pancreas in diabetes is still under investigation. A number of techniques display promise for improving our understanding of diabetes pathogenesis in the pancreas and evaluating response to therapy. The flexibility of MRI, which gives rise to a plethora of techniques interrogating different aspects of disease, is also a hindrance, in that imaging protocols employ different acquisition and processing techniques. Thus, comparing studies across sites can be difficult. Standardized imaging and processing pipelines are needed in order to compare studies and perform multisite clinical trials. Analysis of quantitative MRI studies of the pancreas will be aided by radiomic analysis in order to account for pancreas heterogeneity, especially the heterogeneity characteristic of the diabetic pancreas.

## Author Contributions

The author confirms being the sole contributor of this work and has approved it for publication.

## Funding

This work was supported by JDRF International (3-SRA-2019-759-M-B and 3-SRA-2015-102-M-B) and the Cain Foundation.

## Conflict of Interest

The author declares that the research was conducted in the absence of any commercial or financial relationships that could be construed as a potential conflict of interest.
